# Genome Sequences of *Streptomyces* Phages Amela and Verse

**DOI:** 10.1128/genomeA.01589-15

**Published:** 2016-02-18

**Authors:** Sonya R. Layton, Ryan M. Hemenway, Christine M. Munyoki, Emory B. Barnes, Sierra E. Barnett, Alec M. Bond, Jessi M. Narvaez, Christie D. Sirisakd, Brandt R. Smith, Justin Swain, Orooj Syed, Charles A. Bowman, Daniel A. Russell, Swapan Bhuiyan, Richard Donegan-Quick, Robert C. Benjamin, Lee E. Hughes

**Affiliations:** a Department of Biological Sciences, University of North Texas, Denton, Texas, USA; b Department of Biological Sciences, University of Pittsburgh, Pittsburgh, Pennsylvania, USA

## Abstract

Amela and Verse are two *Streptomyces* phages isolated by enrichment on *Streptomyces venezuelae* (ATCC 10712) from two different soil samples. Amela has a genome length of 49,452, with 75 genes. Verse has a genome length of 49,483, with 75 genes. Both belong to the BD3 subcluster of Actinobacteriophage.

## GENOME ANNOUNCEMENT

A number of *Streptomyces* phages have been isolated from soil on a variety of *Streptomyces* species (1), and various genetic tools derived from *Streptomyces* phages have been valuable in understanding the bacteria and their antibiotic pathways (2). *Streptomyces* phages are grouped into 9 clusters (BA-BI) within the Actinobacteriophages based on nucleotide sequence similarity as well as their gene content (http://www.phagesdb.org). The cluster with the most members, the BD cluster, is divided into 5 subclusters, including BD3.

The BD3 subcluster has three members, including the previously reported phage phiCAM (3) as well as the two phages presented here, Amela and Verse. phiCAM, unlike Amela and Verse, was isolated on *Streptomyces coelicolor*. Amela and Verse were enriched on *Streptomyces venezuelae* (ATCC 10712) from soil samples collected in Frisco, TX and McKinney, TX, respectively. Other *Streptomyces venezuelae* phages are grouped with clusters BA, BC, BD, or they are singletons.

Amela and Verse exhibit a siphoviridae morphotype with approximate head diameter of 60 nm and tail length of 230 nm. DNA was isolated from each and sequenced on an Illumina MiSeq at the Pittsburgh Bacteriophage Institute. Reads from each were assembled using Newbler and Consed software. Each genome assembled into a single contig. Verse had 6,774-fold coverage, and Amela had 2,242-fold coverage. Amela and Verse are dsDNA viruses with genome lengths of 49,452 bp and 49,483 bp, respectively. Both phages have 11 base 3′ extensions with a sequence of 5′-CGGTACGTGAT. The G+C contents of both are 65.6%, and each was found to have 75 protein coding genes. The BD3 subcluster phage have a smaller genome than average for the BD cluster (50,685 bp).

Amela and Verse share many of the same gene functions with each other, as well as with phiCAM. Amela and Verse have 99% average nucleotide identity with one another and 85% with phiCAM. The sequence differences between Amela and Verse are localized primarily to three areas. The region corresponding to Amela bp 639 to 870 contains 66 discrepancies compared to Verse and results in different predicted lengths for gp2 in each. There are also 168 differences in the last 625 bp at the right end of the genomes. The greatest difference between the two genomes is a 138-bp segment beginning at 46,707 of Amela that has no nucleotide similarity with the corresponding region in Verse. This segment is found within Amela_71 and Verse_72 with the predicted proteins sharing 57% amino acid identity. However, Verse_72 shares 93% nucleotide identity and 96% amino acid identity with phiCAM_68.

### Nucleotide sequence accession numbers.

The Amela genome sequence is available from GenBank under the accession number KT186228. The Verse genome sequence is available from GenBank under the accession number KT186229.
